# CADM1 Is a Key Receptor Mediating Human Mast Cell Adhesion to Human Lung Fibroblasts and Airway Smooth Muscle Cells

**DOI:** 10.1371/journal.pone.0061579

**Published:** 2013-04-19

**Authors:** Elena P. Moiseeva, Katy M. Roach, Mark L. Leyland, Peter Bradding

**Affiliations:** 1 Institute for Lung Health, Department of Infection, Immunity and Inflammation, University of Leicester, Leicester, United Kingdom; 2 Department of Biochemistry, University of Leicester, Leicester, United Kingdom; Leiden University Medical Center, The Netherlands

## Abstract

**Background:**

Mast cells (MCs) play a central role in the development of many diseases including asthma and pulmonary fibrosis. Interactions of human lung mast cells (HLMCs) with human airway smooth muscle cells (HASMCs) are partially dependent on adhesion mediated by cell adhesion molecule-1 (CADM1), but the adhesion mechanism through which HLMCs interact with human lung fibroblasts (HLFs) is not known. CADM1 is expressed as several isoforms (SP4, SP1, SP6) in HLMCs, with SP4 dominant. These isoforms differentially regulate HLMC homotypic adhesion and survival.

**Objective:**

In this study we have investigated the role of CADM1 isoforms in the adhesion of HLMCs and HMC-1 cells to primary HASMCs and HLFs.

**Methods:**

CADM1 overexpression or downregulation was achieved using adenoviral delivery of CADM1 short hairpin RNAs or isoform-specific cDNAs respectively.

**Results:**

Downregulation of CADM1 attenuated both HLMC and HMC-1 adhesion to both primary HASMCs and HLFs. Overexpression of either SP1 or SP4 isoforms did not alter MC adhesion to HASMCs, whereas overexpression of SP4, but not SP1, significantly increased both HMC-1 cell and HLMC adhesion to HLFs. The expression level of CADM1 SP4 strongly predicted the extent of MC adhesion; linear regression indicated that CADM1 accounts for up to 67% and 32% of adhesion to HLFs for HMC-1 cells and HLMCs, respectively. HLFs supported HLMC proliferation and survival through a CADM1-dependent mechanism. With respect to CADM1 counter-receptor expression, HLFs expressed both CADM1 and nectin-3, whereas HASMCs expressed only nectin-3.

**Conclusion and Clinical Relevance:**

Collectively these data indicate that the CADM1 SP4 isoform is a key receptor mediating human MC adhesion to HASMCs and HLFs. The differential expression of CADM1 counter-receptors on HLFs compared to HASMCs may allow the specific targeting of either HLMC-HLF or HLMC-HASMC interactions in the lung parenchyma and airways.

## Introduction

Mast cells (MCs) play a primary role in the initiation and propagation of many diseases including asthma and pulmonary fibrosis through the release of numerous proinflammatory and profibrotic mediators [1]. In healthy lungs, MCs are resident in the airway lamina propria and lung parenchyma, but in disease they become activated and redistribute to key tissue structures. In idiopathic pulmonary fibrosis, MCs are in contact with parenchymal fibroblasts [2], with increased numbers of MCs correlating with the degree of fibrosis [3]. In asthma, activated MCs migrate into the airway epithelium [4], airway mucous glands [5] and airway smooth muscle (ASM) [6]. This relocation of MCs within diseased lung tissue facilitates mast cell-structural cell interactions which in turn drives the pathobiology.

Cell-cell adhesion is a fundamental mechanism through which cells communicate, facilitating the delivery of specific cell-cell signals which regulate many cellular processes including proliferation, differentiation, survival and mediator release. With respect to MC heterotypic adhesion to lung fibroblasts (LFs) and ASM cells (ASMCs), there are important bi-directional consequences. For example, direct contact between human lung MCs (HLMCs) and human lung fibroblasts (HLFs)/3T3 fibroblasts or human ASM cells (HASMCs) leads to MC activation and secretion of proinflammatory mediators [7]–[9]. Co-cultures of either fibroblasts or ASMCs with MCs leads to increased production of IL-6 [9], [10]. Moreover, HLMC adhesion to HASMCs induces HLMC proliferation and promotes their survival [9], while adhesion of intestinal MCs to gut fibroblasts results in increased chymase expression and a shift towards the MC_TC_ phenotype [11]. In pulmonary fibrosis, HLMCs develop the MC_TC_ phenotype and their number correlates with accumulation of myofibroblasts expressing α-smooth muscle actin [12].

In turn, mediators released by HLMC in co-culture induce important changes in HASMC and fibroblasts. For example, HLMC adhesion to HASMCs increases HASMC α-smooth muscle actin expression and increases HASMC contractility [13], and direct contact between MCs and fibroblasts increases fibroblast proliferation [14]. In co-cultures with MCs, fibroblasts increase their expression of α-smooth muscle actin and show increased profibrotic responses including enhanced proliferation, migratory activity and collagen production [15]–[18]. Several MC mediators including histamine, tryptase, and IL-4 are responsible for these effects [1], [19]. In summary, cell contact between MCs and either HASMCs or fibroblasts results in MC activation with release of MC mediators, increased proliferation and survival, and a shift to the MC_TC_ phenotype. Conversely, HASMCs and fibroblasts in the presence of MCs develop augmented contractile activity and undergo profibrotic changes.

Identifying the adhesion receptors which facilitate MC interactions with structural lung cells has the potential to identify novel therapeutic targets for the treatment of mast cell-dependent lung diseases. HLMCs adhere to HASMCs in part via cell adhesion molecule 1 (CADM1) which works through a heterophilic molecular interaction [20]. However, the contribution of this adhesion receptor may have been underestimated previously due to the inefficiency of the blocking antibody used [20], [21]. In addition, the α_V_ integrin and CD44 also increase HLMC adhesion to HASMCs in the presence of pro-inflammatory mediators [22]. CADM1 is also implicated in heterophilic adhesion of mouse MCs to 3T3 fibroblasts [23], [24], but the HLMC receptors mediating adhesion to HLFs have not been identified.

CADM1 (also known as IGSF4, TSLC1, Necl-2, SynCAM1) belongs to an immunoglobulin family, which includes CADM2-4, PVRL1-4 (also known as nectin-1-4), PVR (CD155), and CRTAM, as annotated by Ensembl [25]. We have shown recently that CADM1 is expressed as several functional isoforms in HLMCs [26], [27]. Based on the ability to clone CADM1 and western blotting, the SP4 isoform, encoded by exons 1-8/11-12, is the only functional isoform in the mast cell line HMC-1, while HLMCs express ∼80% of SP4, ∼16–18% of longer SP1 (exons 1-9/11-12) and ∼2–5% of the longest SP6 (exons 1-12) isoforms. However, using PCR, mRNA for SP1, SP3 (exons 1–7/11–12), SP4 and SP6 are detectable in both HMC-1 cells and HLMCs [27]. Our studies previously demonstrated differences in survival and homotypic adhesion between MCs overexpressing SP4 and SP1 isoforms [26]. SP1 played a dominant-negative role in survival compared to SP4, and it required longer cell-cell contact to enable homotypic MC adhesion when compared to SP4. CADM1 is involved in adhesion via homophilic and heterophilic interactions with counter-receptors on other cells, and all identified counter-receptors for CADM1 belong to the same family. They include CADM1 itself, CADM2, CADM3, nectin3 and CRTAM [28]–[31]. CD155 binding to CADM1 is controversial [29], [32]. The counter receptors for CADM1 expressed on human ASMCs and HLFs are not known.

The purpose of this work was therefore to investigate: i) whether HLMC adhere to primary HLFs and if such adhesion is CADM1-dependent, ii) whether CADM1 isoforms differentially regulate adhesion of HLMCs to both HLFs and HASMCs, and iii) the expression of counter-receptors for CADM1 in HASMCs and HLFs.

## Materials and Methods

### Ethics statement

All patients donating tissue gave written informed consent and the collection of the various tissues was approved by the National Research Ethics Service (reference 07/MRE08/42) and the Leicestershire Research Ethics Committee (reference 4977).

### Cell Culture

The human MC line HMC-1.1 (V560G) [33] was cultured in IMDM with 10% FBS. HLMCs were isolated from healthy lung obtained at surgery for carcinoma using anti-CD117-coated Dynabeads at ∼99% purity [34]. HLMCs were cultured in DMEM supplemented with 10% FCS, and cytokines (100 ng/ml SCF, 50 ng/ml IL-6, and 10 ng/ml IL-10) as described previously [9].

HASMCs were isolated using explant culture of ASM bundles as previously described [35]. HASMCs were cultured in DMEM supplemented with 10% FBS, antibiotic/antimycotic agents and non-essential amino acids [35]. Parenchymal HLFs were isolated using explant culture from healthy areas of lung tissue obtained at surgery for carcinoma as previously described [36]. HLFs were grown in the same conditions as HASMCs. Each HLF population was stained with anti-fibroblast surface protein 1B10 mAb (F4771, Sigma-Aldrich, Poole ,UK), anti-fibroblast antigen THY-1 mAb (CP28, Calbiochem, Nottingham, UK), anti-α-smooth muscle actin mAb (F3777, Sigma-Aldrich) with positivity of 98%, 97% and 98% (n = 8), respectively. Both HASMCs and HLFs were used at passages 3–5.

### Adenoviral transduction

The CADM1 cDNAs and shRNAs in Ad5C20Att01adenoviral particles (BioFocus, Leiden, the Netherlands) and transduction conditions were as described previously [26]. Adenoviral particles with GFP cDNA and luciferase shRNA (ShLuc) were used as controls. Sh5 alone or a mix of Sh3/Sh4/Sh5, was used for CADM1 downregulation. For adhesion experiments, HMC-1 cells were transduced for 6 days. HLMCs were transduced for 4 days to prevent strong cell-cell aggregation of transduced cells which occurs at 6 days [26], thus ensuring that single cell suspensions were used in experiments.

### Protein analysis

Protein extracts were prepared using mild NP40 (Invitrogen, Paisley, UK) detergent extraction with wide spectrum inhibitors of proteases and protein phosphatases. Protein concentrations of cell extracts were measured using the DC protein assay (Bio-Rad, Hemel Hempstead, UK). SDS–PAGE and immunoblotting were performed using the NuPAGE electrophoresis system (Invitrogen). Whole blots were sequentially probed with antibodies from different species. When possible, blots were cut into horizontal strips with proteins of a particular range of molecular weights, probed with appropriate antibodies. Antibodies against CADM1 (3E1 IgY mAb, Medical & Biological Laboratories, Nagoya, Japan), CADM3 (rabbit IgG, Sigma-Aldrich), nectin-3 (goat IgG, R&D Systems, Abingdon, UK), CRTAM (mAb 16951, R&D Systems), CD155 (mouse mAb SKII.4, Santa Cruz Biotechnology, Germany), Kit (E1, Santa Cruz Biotechnology) and β-actin HRP-conjugated mAb (C4, Santa Cruz Biotechnology, Heidelberg, Germany) were used in this study. Western blots were quantified as previously described [26].

### Flow cytometry

To assess cell surface CADM1 expression following overexpression or downregulation, flow cytometry was undertaken using the CADM1 3E1 IgY mAb as described previously [26].

### Mast cell adhesion

Adhesion assays were performed as previously described [20] with modifications. HLFs or HASMCs were seeded at 10^4^ cells/well in 96 well plates in growth medium. When cells reached confluence after 1–2 days, the medium was replaced with DMEM alone (HLFs) or DMEM supplemented with insulin/transferrin/sodium selenite (ITS-3; Sigma-Aldrich)(HASMCs) for 3 days before use. HMC-1 cells were labelled with 5 µM calcein AM (Invitrogen) according to manufacturer's recommendations prior to adhesion. HMC-1 cells or HLMCs (10^4^ cells/100 µl/well) in DMEM were allowed to adhere to the cell monolayer for 30 min. Wells were then filled with DMEM, plates sealed and then centrifuged upside down at 15g for 5 min. Medium and non-adherent cells were discarded. Adherent HMC-1 cells were detected by fluorescence; adherent HLMCs were detected by histamine assay as previously described [20].

When inhibitory mAbs were used, cells were pre-incubated for 15 min with 10 µg/ml 9D2 IgY mAb against CADM1 (Medical & Biological Laboratories) prior to adhesion assay, as described previously [20].

### HLMC-fibroblast co-culture assay

HLMCs were seeded onto confluent primary HLFs that had been FBS-deprived for 24 h in 6-well plates in DMEM media containing 1% antibiotic/antimycotic, 1% non-essential amino acids and 1% sodium pyruvate. No exogenous growth factors were added. Anti-CADM1 9D2 adhesion-blocking antibody (10 µg/ml) and chicken IgY isotype control (10 µg/ml) were included when required. HLMCs were seeded onto confluent HLFs in 2% FBS at a 1∶4 ratio (equivalent to 1.25×10^4^ HLMCs/well). After 5 days, HLMC numbers were assessed using Kimura staining which readily differentiates red metachromatic mast cells from unlabelled HLFs. HLMC monoculture controls in cytokine-free media containing 2% FBS were established in parallel.

### Data analysis

All experiments were performed independently at least 3 times and at least in triplicate. Analysis was performed using GraphPad Prism 5 software. All data are presented as the mean ± SEM. Differences between two groups were analysed by T-test. Differences among the groups were analysed using a one-way ANOVA, followed by Dunnett's test to determine whether the groups were different from a control group, or Bonferroni's test to compare multiple groups. Regression analysis was used to analyse the effect of CADM1 expression levels on adhesion to HLFs. HLMC counts in co-culture with HLFs were log transformed prior to analysis.

## Results

**Figure 1:**
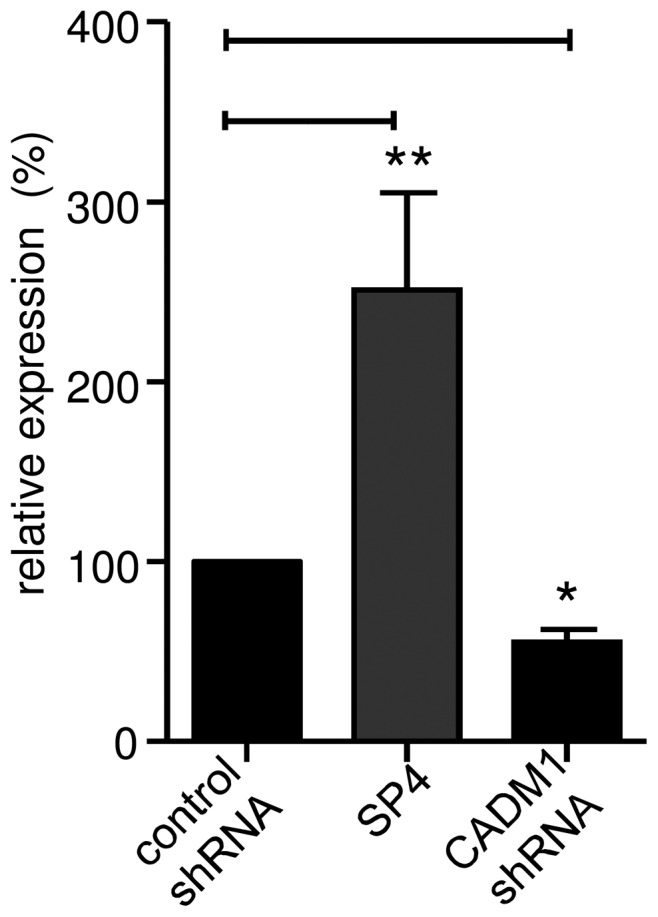
Surface expression of CADM1 in HLMCs following either overexpression of the SP4 isoform or down regulation using shRNA. SP4 cDNA (n = 5) and shRNAs (n = 4) were transduced via adenoviral delivery. p<0.0001 by ANOVA. *p<0.05 compared to luciferase control shRNA, ** p<0.01 compared to luciferase control shRNA (n = 5) using log transformed data.

We have shown previously in parallel experiments performed using the same cells, that adenoviral transduction of HMC-1 cells with CADM1 shRNAs significantly reduced total CADM1 expression to 39±1% and 17±8%, respectively, and surface CADM1 to 41±3% and 25±2%, respectively, of HMC-1 controls [26]. Here, CADM1 downregulation in HLMCs by transduction with shRNA reduced surface CADM1 expression to 55±7% of control after 4 days (Fig. 1).

### CADM1 downregulation reduces HMC-1 and HLMC adhesion to primary human airway smooth muscle cells

**Figure 2:**
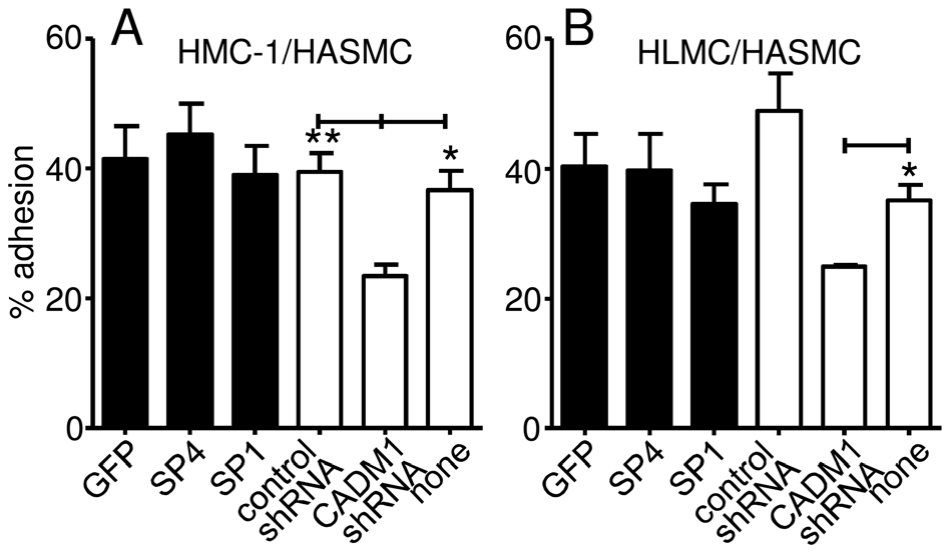
CADM1 is responsible for mast cell adhesion to airway smooth muscle cells. HMC-1 cells and HLMCs were transduced with SP4, SP1, CADM1 shRNA, GFP or luciferase control shRNA using adenoviral delivery, followed by the study of adhesion to HASMCs. A) Downregulation of CADM1 significantly reduced HMC-1 cell adhesion to HASMCs (n = 4), but overexpression of the SP4 or SP1 isoforms did not increase it. *p<0.05, **p<0.01 compared to luciferase control shRNA. B) Downregulation of CADM1 using shRNA reduced adhesion of HLMCs (n = 4) to HASMCs (n = 3, except for luciferase control shRNA where n = 2), but overexpression of SP4 or SP1 did not increase it. *p<0.05 compared to CADM1 shRNA.

Previously we showed that an inefficient adhesion-blocking antibody (9D2) reduced HMC-1 and HLMC adhesion to HASMCs by approximately 20% [20], a likely underestimate of the true CADM1 contribution [21]. Here, downregulation of CADM1 reduced the adhesion of HMC-1 cells to HASMCs from 40±3% in the luciferase shRNA control group or 37±3% in the non-transduced group to 23±2% in the CADM1 shRNA group (P<0.01 and P<0.05, respectively, Fig. 2A). Similarly, CADM1 downregulation reduced HLMC adhesion to HASMCs from 35±2% in the non-transduced control group to 25±0.3% in the shRNA group (P<0.05, Fig. 2B). This confirms our previous findings using an adhesion-blocking antibody [20], but suggests that CADM1 contributes to at least 43% and 29% of the HMC-1 and HLMC adhesion observed, respectively.

### CADM1 downregulation or blocking reduces HMC-1 and HLMC adhesion to primary human parenchymal lung fibroblasts

**Figure 3:**
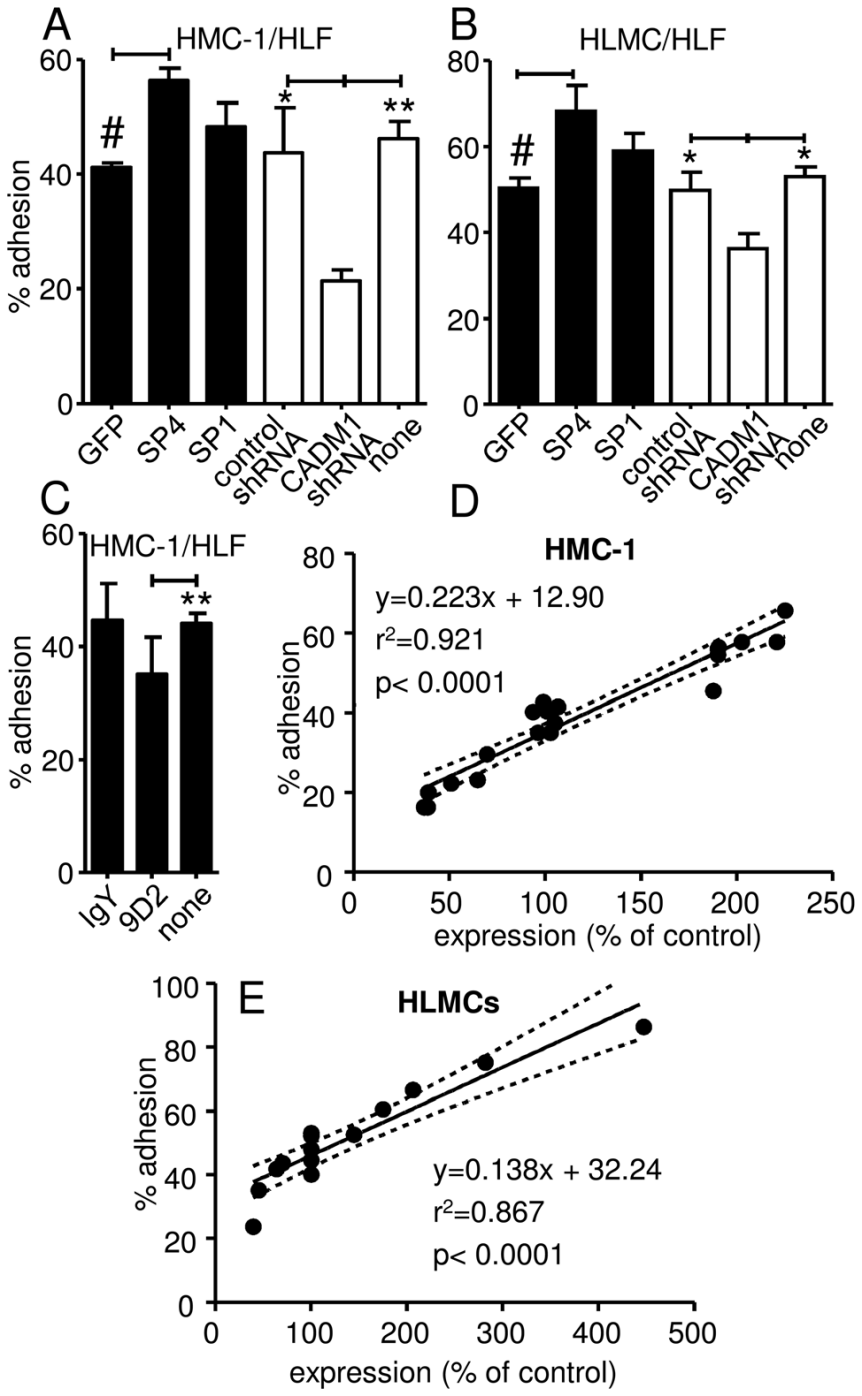
CADM1 is responsible for mast cell adhesion to primary human lung fibroblasts. HMC-1 cells and HLMCs were transduced with SP4, SP1, CADM1 shRNA, GFP or luciferase control shRNA using adenoviral delivery, followed by the study of adhesion to HLFs. A) Downregulation of CADM1 reduced HMC-1 cell adhesion to HLFs (n = 3–6 for different groups), while overexpression of SP4 increased it. *p<0.05, **p<0.01 compared to CADM1 shRNA. #p<0.05 compared to SP4. B) Downregulation of CADM1 reduced adhesion of HLMCs (n = 4) to HLFs (n = 5), while SP4 overexpression increased adhesion. *p<0.05 compared to CADM1 shRNA. #p<0.05 compared to SP4. C) HMC-1 cells pre-incubated with anti-CADM1 9D2 Ab adhered less to HLFs compared to controls (n = 3). **p<0.01 compared to 9D2. D) and E) Scatter plots with regression model parameters for the CADM1 surface expression levels and the degree of adhesion to HLFs by HMC-1 cells (D) and HLMCs (E), which include the data for transduced cells from Fig. 3 including SP4, luciferase control shRNA and CADM1 shRNA transduction groups (20 points in D and 14 points in E) and their expression data published previously in Fig 2b of reference 26 for HMC-1 cells and Fig. 1 in this paper for HLMCs.

The extent to which HMC-1 and HLMC adhere to primary HLFs and the mechanism behind this are not known. CADM1 downregulation reduced adhesion of HMC-1 cells to HLFs from 44±8% in the luciferase shRNA control group or 46±3% in the non-transduced group to 21±3% in the CADM1 shRNA group (P<0.05 and P<0.01, respectively, Fig. 3A). CADM1 downregulation also reduced HLMC adhesion to HLFs from 52±4% in the luciferase shRNA control group or 53±2% in the non-transduced group to 38±4% in CADM1 knockdown group (P<0.05, Fig. 3B). To validate this further, we used an inhibitory anti-CADM1 antibody (9D2), which reduced adhesion of HMC-1 to HLFs from 44±2% in control to 35±6% (P<0.05, Fig. 3C). This also shows that the inhibitory antibody did not inhibit adhesion as efficiently as RNA interference.

Overall, CADM1 downregulation reduced HMC-1 cell adhesion to both HASMCs and HLFs by approximately 50%, while adhesion of HLMCs was decreased by approximately 30% for both HLFs and HASMCs.

### CADM1 SP4 overexpression using adenoviral transduction increases adhesion to lung fibroblasts but not to airway smooth muscle cells

We have shown previously in parallel experiments performed using the same cells, that adenoviral transduction of HMC-1 cells with the SP4 and SP1 CADM1 isoforms markedly increases surface CADM1 to 206±10% and 148±4%, respectively, compared to controls [26]. Similarly SP4 overexpression here in HLMCs increased surface CADM1 expression to 251±121% of control after 4 days (Fig. 1).

Adhesion of HMC-1 cells to ASMCs was very similar for GFP control, and SP4- and SP1-transduced HMC-1 cells (40±5%, 45±5% and 39±5%, respectively, Fig. 2A). Similarly, adhesion of HLMCs to HASMCs was 40±5%, 40±6% and 35±3% for the GFP, SP4 and SP1 groups, respectively (p>0.05, Fig. 2B). Hence, overexpression of either SP4 or SP1 in either HMC-1 cells or HLMCs using adenoviral transduction did not affect adhesion to HASMCs. This suggests that CADM1-dependent adhesion of MCs to HASMCs is maximal at baseline, with all counter-receptors on HASMCs engaged.

In contrast, SP4 overexpression in HMC-1 cells increased adhesion to HLFs from 41±1% in the GFP control group to 56±2% in the SP4 group (P<0.05, Fig. 3A). Similarly, SP4 overexpression increased adhesion of HLMCs to HLFs from 50±2% in the GFP control group to 68±6% in the SP4 group (P<0.05, Fig. 3B). SP1 isoform overexpression also increased adhesion of HMC-1 cells (48±4%) and HLMCs (59±4%) to HLFs when compared to GFP controls, but these increases did not reach statistical significance. These results suggest that unlike HASMCs, additional CADM-1-binding capacity is present on HLFs.

### Regression analysis indicates CADM1 mediates up to 67% of the adhesion of HMC-1 cells to human lung fibroblasts

Our data indicate that the higher the levels of overexpression of either SP4 in HMC-1 cells or HLMCs, the more cells adhere to HLFs. However, while CADM1 downregulation in HMC-1 cells or HLMCs by RNA interference was very effective [26], it was not complete, suggesting that we may have underestimated the contribution of CADM1 to MC adhesion. To examine this further, we compared the data for the surface expression of CADM1 and the adhesion of HMC-1 cells to HLFs. The levels of CADM1 surface expression correlated strongly with the extent of adhesion to HLFs (P<0.0001, Fig. 3D). Furthermore, regression analysis showed that if CADM1 expression was to equal 0, HMC-1 cell adhesion to HLFs would fall to 13%. Hence, CADM1 in HMC-1 cells is a major adhesion receptor, which accounts for up to 67% of their net adhesion to HLFs. Similarly, linear regression analysis of HLMC adhesion demonstrated a strong correlation with the levels of CADM1 expression in these cells (P<0.0001, Fig. 3E). This suggests that CADM1 accounted for 32% of total HLMC adhesion to HLFs.

### Counter receptors for CADM1 on human lung fibroblasts and airway smooth muscle cells are different

**Figure 4:**
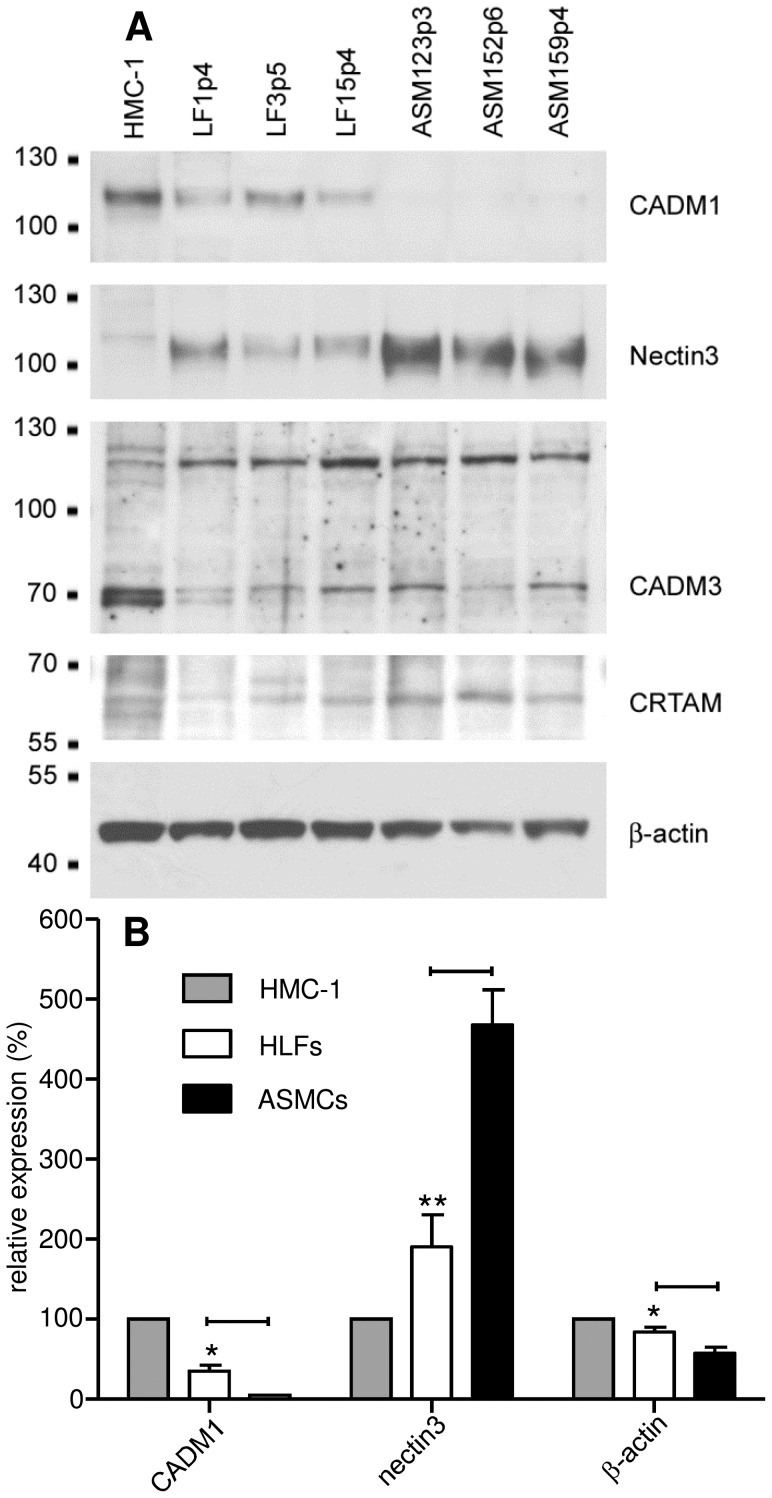
CADM1 counter-receptors in parenchymal human lung fibroblasts and airway smooth muscle cells. A) Western immunoblot of proteins (25 µg/lane) from HLFs and HASMCs from 3 donors each or HMC-1 cells for CADM1 counter-receptors (shown on the right) with molecular markers (shown on the left). B) Quantification of CADM1, nectin-3 and β-actin levels in HLFs and HASMCs relative to HMC-1 cells. *p<0.05, **p<0.01 compared to ASMCs.

CADM1 is involved in adhesion via interactions with counter-receptors on other cells. We therefore compared the expression of known CADM1 counter-receptors in HLFs and HASMCs using western blotting (summarised in Fig. 4). Both cell types expressed low levels of CRTAM and CADM3, which were detectable only after prolonged film exposures. CADM3 was present as both a monomer and a dimer. PVR (CD155) was not detectable (not shown). Nectin-3 was also expressed in HMC-1 cells. Nectin-3 was expressed at higher levels in HASMCs than HLFs: 468±75% and 190±70% of that in HMC-1 cells (p<0.01 for ASMCs compared to HLFs, Fig. 4B). In contrast, CADM1 was expressed in HLFs although at lower levels than in HMC-1 cells (35±13% compared to HMC-1 cells), but it was barely detectable in HASMCs at the same film exposures as for HLFs (5% compared to HMC-1 cells)(p<0.05 for HASMCs compared to HLFs, Fig. 4B). Interestingly, the less CADM1 was expressed in HLFs, the more nectin3 was found in them (compare LF3 with LF1 and LF15 in Fig. 4A). Expression of β-actin also varied in these cell types (58±12% in HLFs and 84±10% in HASMCs compared to HMC-1 cells), but not as much as expression of CADM1 or nectin-3. No reliable antibodies were available for CADM2. These expression data are consistent with the relative mRNA levels of expression of CADM1, CADM3 and nectin-3 reported for HLFs and various smooth muscle cells in the NCBI record GDS1402 [37]. Similar relative mRNA expression levels (CADM1>nectin3>>CADM3, CADM4, CRTAM; [CADM2 is not available]) are also reported in human embryonic fibroblasts in the NCBI record GDS2445 [38]. The HMC-1 cells expressed more CADM1 protein than CADM3 or nectin-3 in agreement with mRNA levels in HLMCs, which express CADM1>>CADM3 or PVR, but not nectin-3, CADM4 or CRTAM; CADM2 is not available [39]. Collectively, human MCs expressed CADM1 at the highest level among this group of interacting adhesion receptors, whereas HASMCs expressed predominantly nectin-3, while HLFs expressed both CADM1 and nectin-3.

In order to investigate if CADM1 expressed in HLFs is involved functionally in adhesion to MCs, we modulated CADM1 using adenoviral transduction. However, expression of CADM1-GFP and SP4 resulted in rounding of cell aggregates and their detachment. Downregulation of CADM1 with shRNA resulted in peeling cells from the plastic surface. In both cases, modulation of CADM1 in HLFs resulted in destroyed fibroblast layers, which prevented their use in adhesion assays.

### CADM1 promotes lung fibroblast-dependent HLMC survival and proliferation

**Figure 5:**
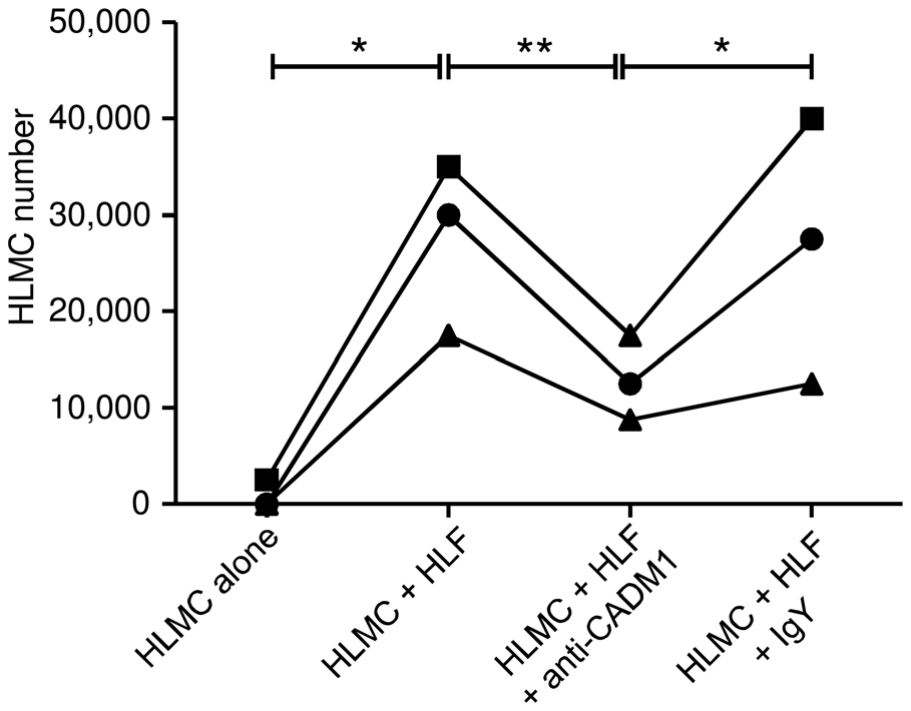
CADM1 promotes lung fibroblast-dependent HLMC survival and proliferation. HLMC numbers (n = 3 donors) after 5 days culture in media alone, or co-culture with HLFs (n = 3 donors). CADM1-adhesion blocking antibody or isotype control were added where indicated. 12500 HLMCs were present at initiation of the cultures. *p<0.05, ** p<0.01. Statistics performed on log transformed data, zero values for cell counts given nominal value of 100 for log transformation.

When HLMCs (1.25×10^4^)(n = 3 independent donors) were incubated in culture medium alone their numbers diminished to almost zero by day 5 (Fig. 5). When HLMCs (n = 3) were co-cultured with HLFs (n = 3 independent HLF donors) (4∶1 HLF∶HLMC ratio), not only did they survive, there was rapid proliferation evident by day 5 with the number of HLMCs increasing to 2.75±0.52×10^4^ (n = 3, p = 0.041 compared to cells alone; Fig. 5). Incubation with CADM1 adhesion-blocking antibody significantly reduced the number of HLMCs in co-culture when compared to no-antibody control (p<0.01, Bonferroni's post test) or IgY isotype control (p<0.05, Bonferroni's post test)(p = 0.0067 across groups by repeated measures ANOVA)(Fig. 5). These data show that CADM1-mediated adhesion to HLFs not only supports HLMC survival, but rapidly promotes HLMC proliferation.

## Discussion

Here we demonstrate for the first time that CADM1 is an important receptor mediating HLMC adhesion to primary HLFs, and that CADM1-dependent adhesion to HLFs promotes HLMC survival and proliferation. We have also confirmed its importance for adhesion to HASMCs. However, the profile of CADM1 counter-receptors on HLFs and HASMCs differ, which is likely to account for the different capacity for MC-associated CADM1 binding on these two cell types.

Surface CADM1 downregulation in HMC-1 cells and HLMCs, to ∼20% and 55% respectively, reduced their adhesion to HASMCs and HLFs by ∼50% and ∼30%, respectively. CADM1 inhibition by an inhibitory antibody reduced MC adhesion to HLFs by ∼25%, and in a previous study, this antibody reduced MC adhesion to HASMCs by ∼22% [20]. These results suggest that the inhibitory antibody is less efficient in inhibiting adhesion than RNA interference, which is in keeping with the ability of the antibody to only reduce aggregation of CADM1 transfectants by approximately 50% rather than 100% [21]. In fact, regression analysis indicated that CADM1 is responsible for about 67% and 32% of HMC-1 and HLMC net adhesion to HLFs, respectively. Hence, CADM1 involvement in adhesion of MCs was significantly higher in neoplastic HMC-1 cells compared to differentiated HLMCs, which may reflect the 4-fold higher surface CADM1 expression levels in HMC-1 cells compared to HLMCs as shown previously [26]. CADM1 is therefore an important adhesion receptor for the adhesion of HMC-1 and HLMC to both HASMCs and primary HLFs. The physiological relevance of this is underpinned by the observation that in healthy lung parenchyma, 50% of the HLMC surface is found in direct contact with HLFs [2].

Identification of the remaining CADM1-independent constitutive adhesion mechanism between HLMCs and HASMCs/HLFs requires further investigation. This is likely to include integrins and other adhesion receptors for the extracellular matrix, since both HLFs and HASMCs produce and assemble significant amounts of extracellular matrix on the cell surface. Indeed, HLMC adhesion to HASMCs is partially reduced by EDTA [20], although none of the common β1 or αv integrins expressed on HLMCs appeared to be involved in the constitutive adhesion of unstimulated cells [20]. However, the integrin activator Mn^2+^ did increase HLMC adhesion to HASMCs, and the α_v_ integrin and CD44 were involved in the increased adhesion of HLMCs to HASMCs following activation with TGFβ1 and TNFα [22]. Nonetheless, our data indicate that CADM1 has a major effect on HLMC adhesion to structural human lung cells and consequently on CADM1-mediated proliferation of HLMCs in co-culture.

Transduction of CADM1 SP4 in both HMC-1 cells and HLMCs dramatically increased adhesion to HLFs, but not ASMCs, suggesting differential regulation of the adhesion to these two cell types. Interestingly, the CADM1-counter receptors in HLFs and HASMCs proved to be different. Nectin-3 was the predominant CADM1 counter-receptor in HASMCs, whereas both CADM1 and nectin-3 were highly expressed in HLFs. Both cell types also expressed low levels of CADM3 and CRTAM. Hence, MC CADM1-dependent adhesion to HASMCs is heterophilic, whereas adhesion to HLFs appears to be partially homophilic. The expression of CADM1 by human HLFs is interesting because CADM1 was not expressed by dedifferentiated mouse 3T3 fibroblasts, and mouse MC CADM1-dependent adhesion to these cells was therefore heterophilic [23]. These observations again highlight important tissue- and species-dependent heterogeneity with respect to cell biology.

While SP4 transduction increased MC adhesion to HLFs, the same was not true for SP1. In part this may be because surface CADM1 expression increased more with SP4 transduction than SP1. However, SP1 was still upregulated by about 50% in transduced cells [26]. This observation is in keeping with the model we have proposed previously regarding the potential effect of mixed isoforms on cell adhesion [26]. SP4 is the only functional isoform expressed in HMC-1 cells, and the dominant isoform in HLMCs, so overexpression of SP1 leads to the presence of mixed isoforms. Dimerisation is important for CADM1-dependent adhesion, and since SP1 has a longer extracellular domain than SP4 [40], it is unlikely that it can dimerise efficiently with the shorter SP4; the consequence of this would be slower dimerisation of SP4/SP4 and SP1/SP1 compared to faster dimerisation of SP4/SP4 in cells expressing only this isoform, and hence less cell-cell adhesion.

A question arises about the biological reason for the differential expression of CADM1 isoforms in HLMCs. Both SP1 and SP6 contain a TTATTEPAVH sequence, encoded by exon 9, with a recognition site for a non-identified protease [41], and they can be shed from the cell surface. Thus, adhesion via these isoforms can be regulated. Although speculative, if these isoforms are shed from the cell surface of stably adherent cells, the strength of adhesion would be reduced. Alternatively, if these isoforms are shed from the cell surface of non-adherent cells, the adhesive potential of cells may increase by the presence of a single SP4 isoform, which also provides pro-survival signalling [26]. Therefore, expression of various isoforms with a predominance of SP4 in HLMCs may provide a regulatory mechanism for cell adhesion and viability.

Our data demonstrate an important functional consequence mediated by CADM1-dependent adhesion of HLMCs to primary HLFs. Fibroblasts not only maintained HLMC survival in the absence of exogenous growth factors, but also induced HLMC proliferation as described previously in primary HASMCs [9]. This was significantly inhibited by CADM1 adhesion-blocking antibody. This suggests that CADM1 may be a key molecule regulating the HLMC-HLF cross-talk which is believed to be important in the progression of idiopathic pulmonary fibrosis [1], and as such may prove to be an interesting therapeutic target.

In summary, our data show that CADM1 is a key adhesion receptor mediating the adhesion of HLMCs to primary HLFs and HASMCs, but that this occurs via interactions with different counter-receptors on these cell types. The SP4 isoform expressed on HLMCs appears to mediate cell-cell adhesion more efficiently than SP1. Cell signalling driven by CADM1 in HLMCs and its counter-receptors in HASMCs and HLFs is likely to initiate important biological effects which result from the adhesive interactions which occur between these cell types. Targeting these signalling pathways may offer new therapeutic approaches for the treatment of asthma and pulmonary fibrosis.
